# Tracing Road Network Bottleneck by Data Driven Approach

**DOI:** 10.1371/journal.pone.0156089

**Published:** 2016-05-26

**Authors:** Hongsheng Qi, Meiqi Liu, Lihui Zhang, Dianhai Wang

**Affiliations:** College of Civil Engineering and Architecture, Zhejiang University, 866, Yuhangtang Road, Hangzhou, 310058, China; Chongqing University, CHINA

## Abstract

Urban road congestions change both temporally and spatially. They are essentially caused by network bottlenecks. Therefore, understanding bottleneck dynamics is critical in the goal of reasonably allocating transportation resources. In general, a typical bottleneck experiences the stages of formation, propagation and dispersion. In order to understand the three stages of a bottle neck and how the bottleneck moves on a road network, traffic flow data can be used to reconstruct these dynamics. However, raw traffic flow data is usually flawed in many ways. For instance some portion of data may be missing due to the failure of data collection devices, or some random factors in the data make it hard to identify real bottlenecks. In this paper a “user voting method” is proposed to deal with such raw-data-related issues. In this method, road links are ranked according to the weighed sum of certain performance measures and the links that are ranked relatively high are regarded as recurrent bottlenecks in a network, and several bottlenecks form a bottleneck area. A series of bottleneck parameters can be defined based on the identified bottleneck areas, such as bottleneck coverage, bottleneck link length, etc. Identifying bottleneck areas and calculating the bottleneck parameters for each time interval can reflect the evolution of the bottlenecks and also help trace how the bottlenecks move.

## Introduction

Traffic congestion often occurs at or originates from bottleneck areas. Therefore, modeling and analysis of traffic bottleneck are crucial for relieving traffic congestion and improving transportation operation. When bottlenecks propagate in the network, the network operational efficiency would be greatly decreased [1, 2].

Literature on traffic bottlenecks can be found in fields like transportation economies, physics of traffic flow and traffic engineering [3]. The majority of the work in transportation economies focuses on congestions induced by bottleneck during morning commute time. The “bottleneck model” formulated by Vickrey [4] is a fundamental model in congestion analysis. The model provided significant insights for understanding many features of traffic congestion. Many follow-up research papers extended the “bottleneck model”, e.g. Xiao et al [5] considered the case that two flows merged into one link, and the link capacity was stochastic. In physics, traffic bottleneck studies usually focus on local-level bottlenecks. For example, in Zhang et al.[6], the bottleneck was caused by lane drops; Nakata et al. [7] studied the game dilemma at a two into one bottleneck junction; in a two-route system, Hino and Nagatani [8] derived the travel time and mean density according to the bottleneck’s strength; Kerner et al. [9] proved the nucleation nature of empirical traffic breakdown at highway bottlenecks with traffic flow data of nearly 20 years. From the perspective of traffic engineering, much effort has been devoted to analyze the characteristics of bottlenecks and the control of bottlenecks. Bottlenecks can be either dynamic or static. Schrank et al. [10] described how to extract 100 most congested roadway sections, based on velocity and volume data; Seeherman and Skabardonis [11] analyzed the effect of weather on the recurrent delay at two types of freeway bottlenecks, one incurred by traffic merge and the other by lane drop; Li et al. [12] used a logic-tree based method to adjust the speed of a bottleneck at Auckland; Sun et al. [13] studied the mechanism of the formulation of expressway bottlenecks, and their focus was mainly on the lane changing behaviors around the bottleneck; John et al. [14] used speed data to report the bottlenecks of the nation-wide road network. The slope of the fitted line between the speed vectors of any pair of neighbored links was used to describe the trend of traffic congestion; Chen and Ahn [15] investigated the variable speed limit control of non-recurrent bottlenecks.

The above studies provide various methods to analyze freeway bottlenecks, which may not be directly applicable to the urban street network, since urban street system operates different from freeway system. Without a mainstream-merge-diverge structure, it is hard to decide where the bottlenecks are located. All these make it difficult to analyze the dynamics of bottlenecks in the urban street network, which is the reason of why urban network bottleneck models are lacking.

Due to the development of intelligent transportation system, various types of traffic flow data become available, making the analysis of bottlenecks with a data-driven approach possible [16]. However, due to various reasons, the raw data usually exhibits many problems. For example, some portion of the data may be missing, due to unavailability of detectors or failures of data collection devices. Some random factors, such as incident and temporal parking, can also bring in noises which make it unreliable to directly derive the bottleneck dynamics from the raw data.

**Fig 1:**
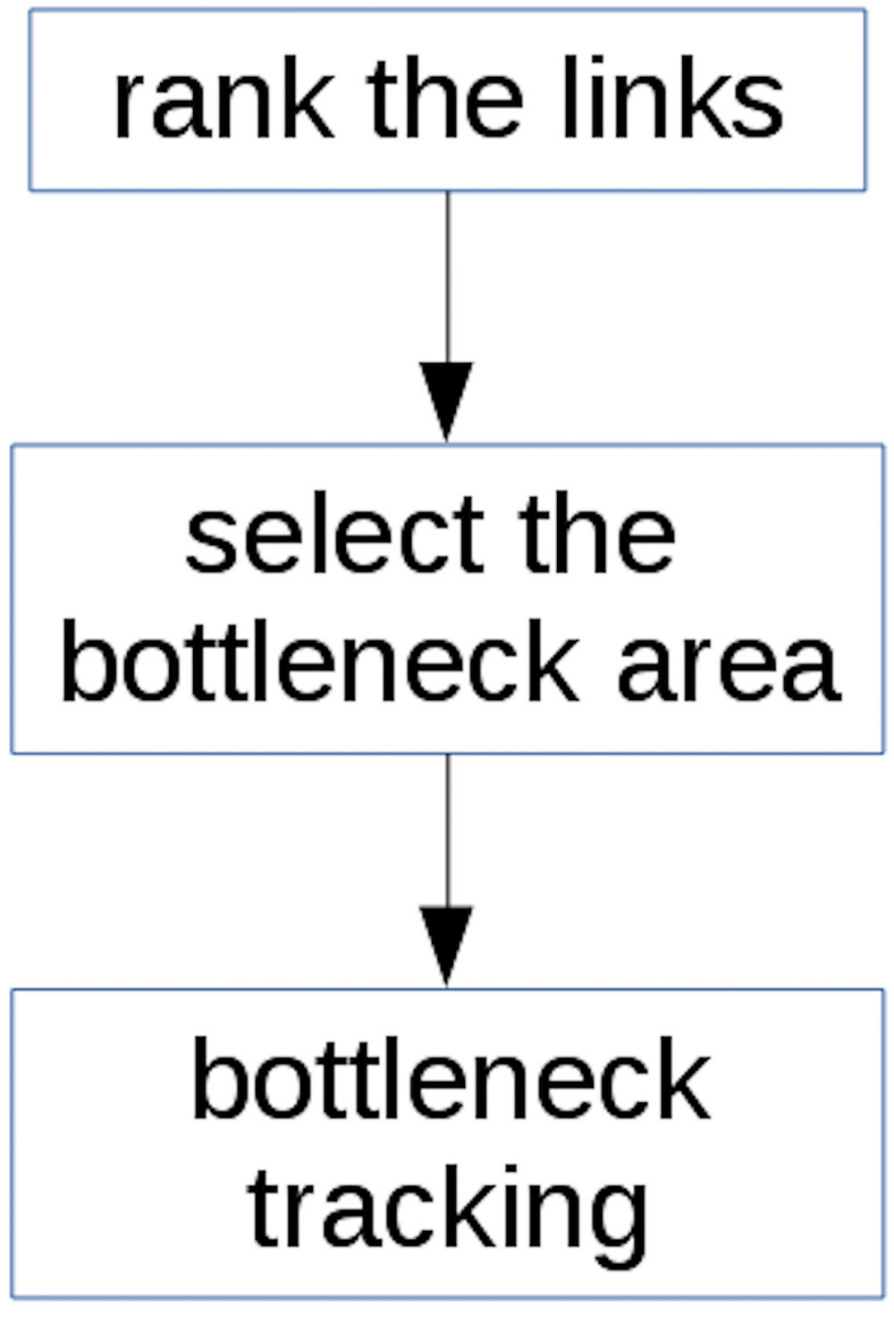
Workflow of the model.

Due to numerous random factors in the transportation system, it may not be easy to directly recognize the bottlenecks from large scale traffic data, since the data themselves is stochastic in nature. To deal with the uncertainties of the link performance measures, this paper introduces the method of voting and ranking. In the typical ranking problem, a lot of goods are voted by many users. The eventual rank of the good is derived based on the weighed sum of all voted scores, taking the random factors of the users' preferences into account. Similarly, the ranking of the performance of a specific link for each time interval can also be regarded as a “score”. The ranking of a link is determined by the weighed sum of the velocity data of the link. Links ranks higher than certain criteria can be considered as potential bottlenecks. Analyzing the time dependent link rankings can help track bottlenecks. Fig 1 describe the workflow of the method.

The rest of the paper is structured as follows: the next section describes the data set used in our analysis; following that the ranking models as well as the bottleneck selection method are given in Section 3; Section 4 presents some numerical results to demonstrate the validation of the proposed model. Conclusions are given in the last section.

## Data Descriptive

### Data Source

The data includes two types: the geographical data set and the velocity data set. The geographical data is in a shapefile format which is developed and regulated by Esri (Environmental Systems Research Institute). This data set helps to visualize the traffic state in the network. The vehicle velocity data is the taxi GPS data collected from 2012-11-01 to 2012-11-30 in city of Hangzhou, China, included in the S1 File. There are totally around 9000 taxis in the city. The GPS equipments send second by second location and travel direction information to the traffic management center. The location can be matched to the geographical data to determine which link the taxi is driving on. In this way, the travel trajectory of each vehicle can be obtained. To calculate the velocity of each link, the entire modeling time horizon is divided into intervals of five-minutes. For a specific link *i* at time interval *k*, vehicles traversed the link is selected from raw data. Suppose that, for a vehicle selected within this time interval, the initial spatial-temporal coordinate is (*t*_1_, *x*_1_) and the final coordinate is (*t*_2_, *x*_2_), then the mean velocity within this spatial-temporal space can be calculated as $\frac{x_{2}-x_{1}}{t_{2}-t_{1}}$. These mean velocities are then averaged over all the vehicles to obtain the link velocity. For each link, there will be totally 288 successive velocities within an analysis period of 24 hours. The directional velocity is not considered. But for the description of the whole network, such simplification is acceptable. A two-way street is modeled as two links here, and the velocity is calculated separately for each link.

**Fig 2:**
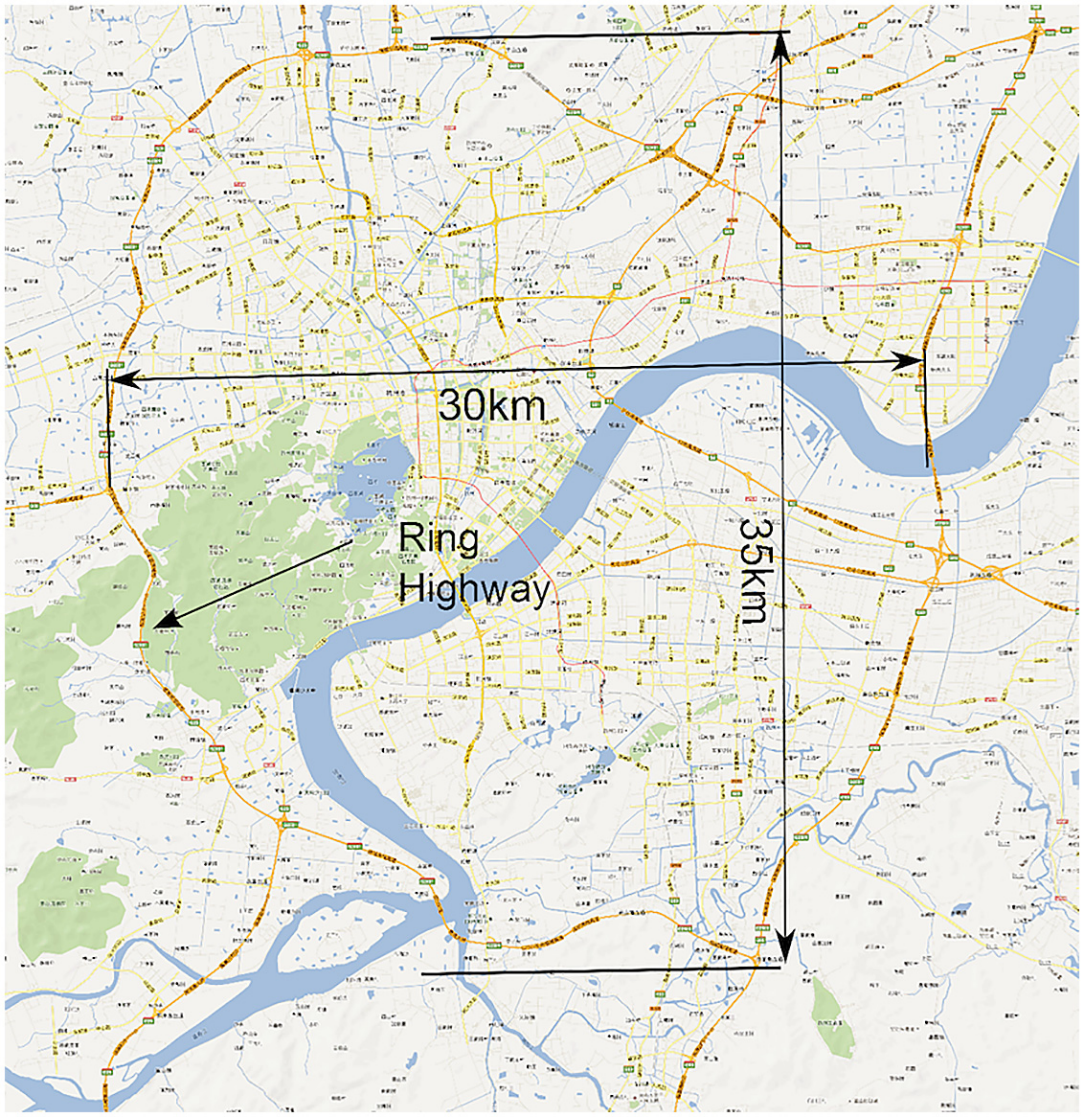
Data coverage area of HangZhou city.

**Fig 3:**
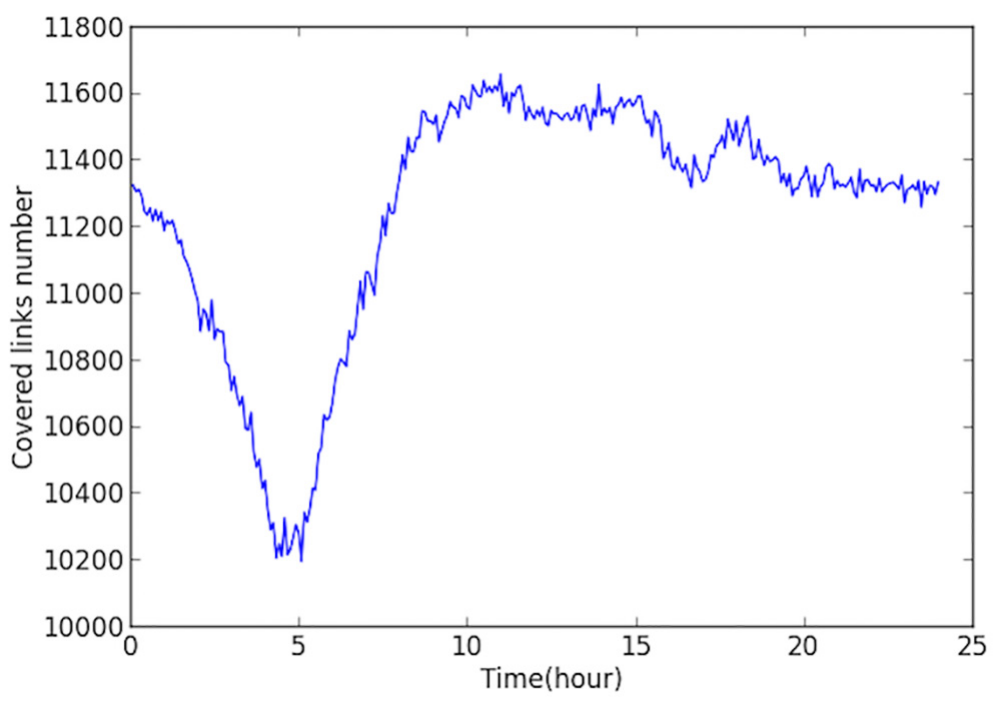
Covered links number VS time (2012-11-01).

The studied network covers all the links within the “Ring Highway” of Hangzhou City, as shown in Fig 2. The horizontal and vertical boundary distances are about 30km and 35 km respectively. Within this area, there are about 13402 directional links. Taxis may not use some links during some time intervals, and Fig 3 shows the numbers of links covered by taxi trajectory during different times of a day. Since the majority of the roads velocity is available, and the number of links is relatively low in the early morning, and we mainly focus on the peak hours rather than off-peak hours, such coverage is acceptable.

### The Temporal Profile of the Whole Network

**Fig 4:**
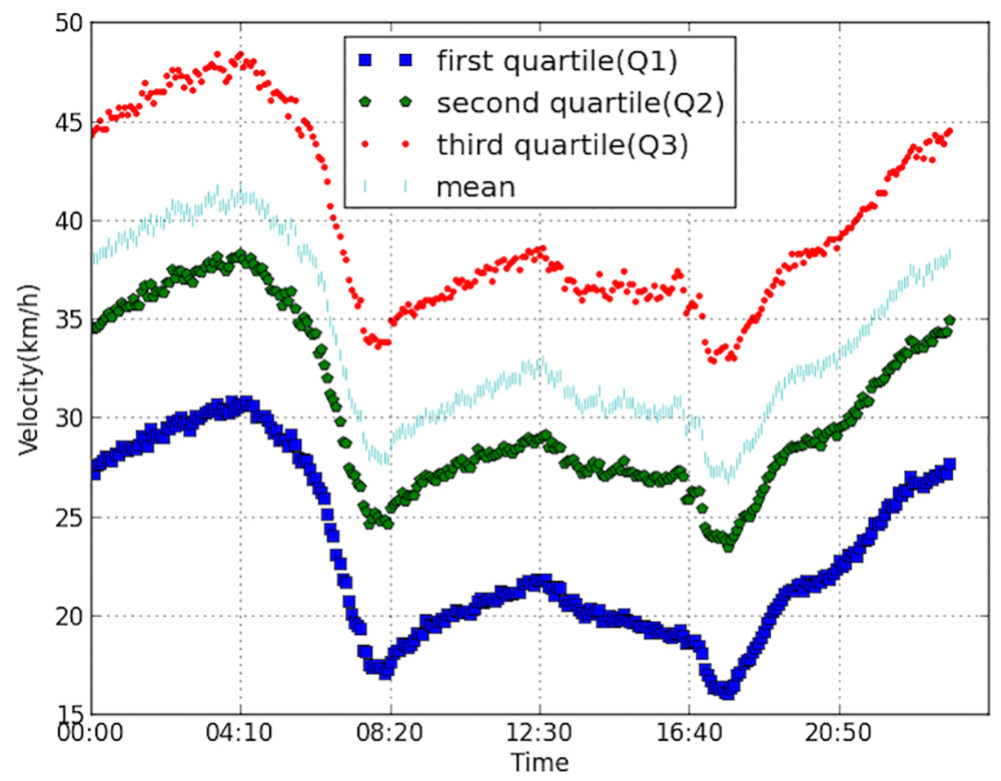
Velocity profile of the whole network.

Fig 4 shows the velocity profile of the whole network, where four statistics are presented, s including the mean, the first, second and third quartiles of the velocity. The four statistics exhibit the same trend. In the early morning, velocity increases and reaches the peak around 04:00 and decreases sharply afterwards till about 08:00. The velocity during the day time is generally smaller and the peak appears around 12:30. The evening congestion period of Hangzhou City normally last from17:00 till 19:00. As can be observed from the figure, during this period a majority of the vehicles go below 30 km/h, and among them quite many goes below 20 km/h.

### Links under Different LOS

Level of service (LOS) is an intuitive measure for traffic flow operation. Highway capacity manual defines different LOSs according to link velocities. The detailed definitions are given in Table 1.

**Table 1:** Definition of LOS.

Velocity	>50	(39,50]	(28,39]	(22,28]	(17,22]	<= 17
LOS	A	B	C	D	E	F

**Fig 5:**
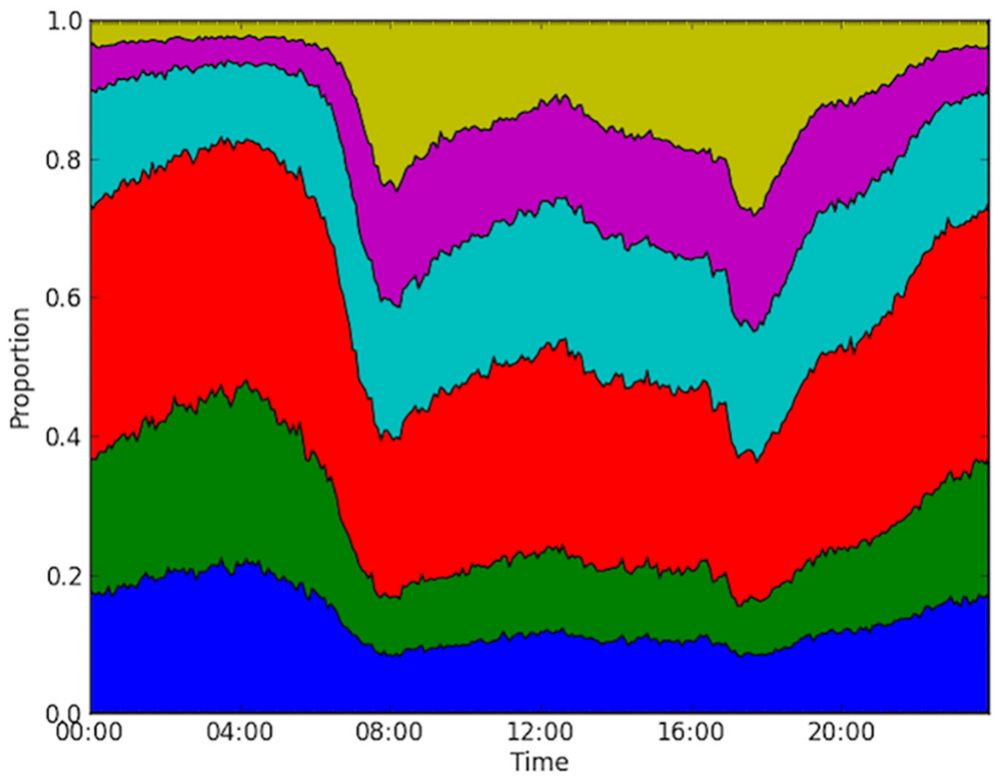
Proportions of Links under different LOS.

Fig 5 shows the proportions of links under different LOSs From the bottom up, the LOSs are A, B, C, D, E, and F respectively. The coverage of LOS A is relatively stable, while that of LOS F changes sharply during the morning and evening congestion periods, when the coverages are both over 20% of the whole network. The coverage of LOS A does not change dramatically since these links are mainly rural highway links, which are less influenced by commuting traffic. Among all the LOSs, the LOS C always covers the largest number of links.

### Traffic State for Different Road Types

Roads of different types operate substantially differently. Major road types in the study area include principal arterials, minor arterials, principal branches, minor branches, expressways and highways.

**Fig 6:**
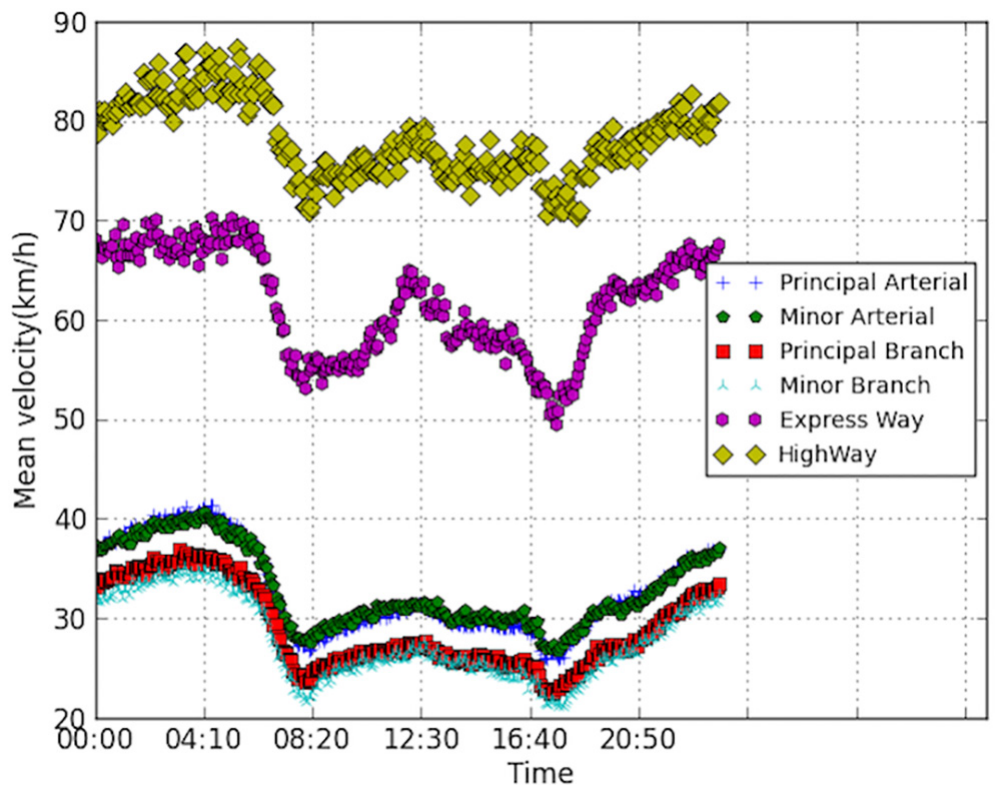
Velocity profiles of different road types.

Fig 6 presents the mean velocities of different road types. The velocity is averaged across all links of the same type. The performances of expressways are obviously better than other types, while velocities of the branch roads are relatively smaller. Principle and minor arterial roads perform slightly better than the branch roads. The temporal profiles of different road types present similar trend, with same trough hour.

**Fig 7:**
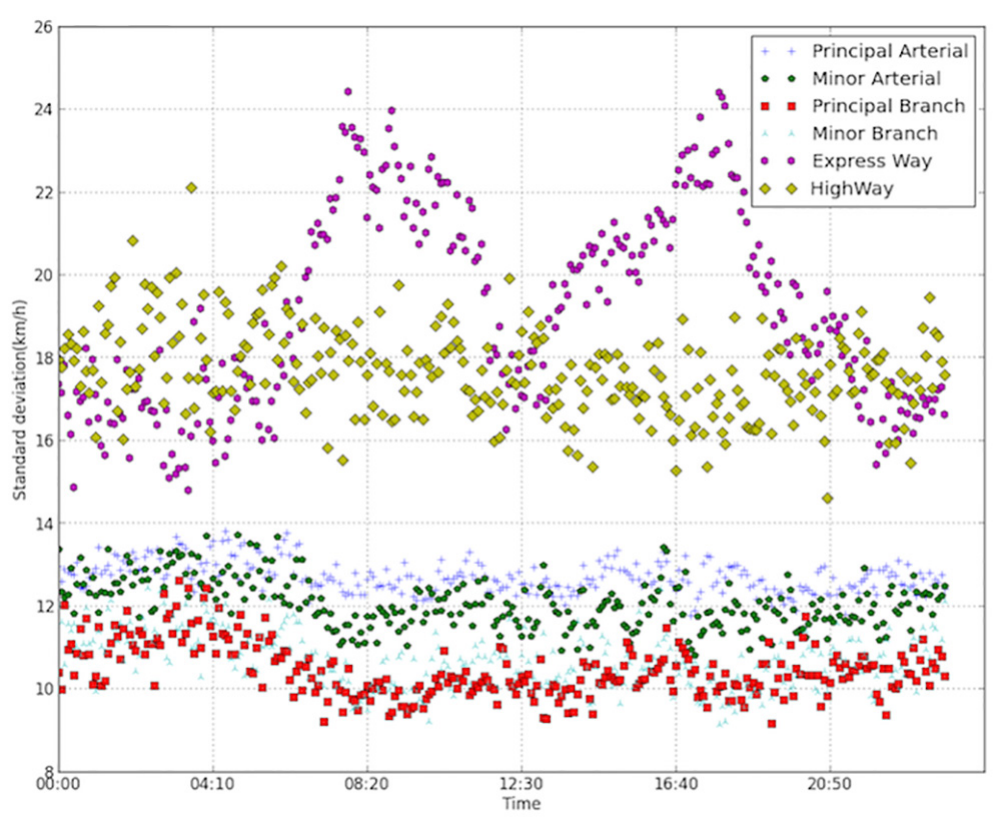
Standard deviations of the velocities for different road types.

Fig 7 displays the standard deviations of the velocities for different road types. The deviations are computed across all links of the same types within the same time interval. Interestingly, the deviations of most road types remain relatively stable during a whole day, except that of the expressway. The reason may be attributed to the function of the expressway. Expressway serves the traffic flowing in and out of the city. The arterial and branch roads mainly serve the commuters within the city. When morning/evening congestion peak approaches, part of the expressway system is also influenced by the daily commuting traffic, while the remaining part stays uninfluenced, which leads to a large variation of the velocity. The capacities of expressways are generally larger than those of surface streets, making them more attractive to travelers during congested periods. Thus when peak hour comes, drivers may divert to expressways even though at the cost of longer travel distance. The coincidences of larger standard deviations and smaller mean velocities during peak periods show that expressway system plays as a buffer role in accommodating daily traffic.

## Model

### Rank of the Traffic State

We first define the following terms:

1. score: the evaluation result of each link by each time interval. In our analysis, the velocity is regarded as a type of score;
2. rank: the relative congestion level of a link in the road network. We use the parameter velocity to denote the traffic state, and a higher rank implies a more congested traffic state;
3. bottleneck links: based on some congestion threshold, a series of links which rank top in the network can be selected. These connected bottleneck links form a bottleneck area. Subsequently, bottleneck parameters can be defined, like coverage, center, etc.

The basic idea of ranking is that during some time domain, the relative traffic state of a link should be stable, which can be represented by the rank of a reference parameter, such as velocity, among all the links. The rank of a link in each time interval within the time domain can be seen as a “realization” of the rank hence operates at a random manner. It is assumed the worst performance ranks the first, thus the bottleneck links will be ranked at the top, either because the velocities of these links are relatively smaller or the travel times on these links are larger. It is supposed that all velocity data associated with this link contribute to the rank, with different weighting factors. The rank can be obtained according to the weighed velocity, or called score here.

The above description is similar to the user voting problem, in which a lot of users vote for a series of goods. Each user will evaluate one or more goods and give a score to each of them. Due to the user preferences and other uncertain factors, the scores for a good essentially form a distribution. The final evaluation of the good is an aggregation of all these scores. We implement the voting process in a recursive way. During each iteration, the weight of each velocity is updated, until some indexes are within a predefined threshold.

By referring to a term “time domain”, we want to track the evolution of bottlenecks in a network along the time horizon. For example, we first analyze the velocity data of one hour, and the bottleneck links within this hour can be identified. Then we roll ahead the time domain by 10 minutes and analyze the corresponding one-hour velocity data again, and the bottleneck links during this hour can also be recognized. The new set of bottleneck links may not be the same, due to the change of network demand and supply. By performing the same analysis for each one-hour domain, the evolution of bottlenecks can be tracked. For instance, whether the bottleneck area expands or shrinks, or shifts to other locations.

#### Model description

*v*_*ij*,*k*_ is used to denote the velocity of link *k* during time interval *j* of day *i*. Scores by intervals in peak hours are more reliable than off-peak hours. The weighting factors of the scores are determined based on the following rules: 1) the weight is larger for peak hour intervals; 2) the rank of a link during one interval should be similar to all other intervals. The rank of a link during one interval can be different from its ultimate rank, which represents the overall congestion degree.

We relate the weight of a velocity data of a link during certain interval to three factors:

1. the average velocity of the whole network during that time interval;
2. the discrepancy between the rank derived according to this data and the rank derived according to the ultimate score;
3. the discrepancy between the rank given by that interval and the ranks given by all other intervals;

Some notations are first given as follows:

*s*_*k*_ ∈ (0,1]: the ultimate score of a link *k*, *S* = {*s*_*k*_} is the vector for all links;

θ_*ij*,*k*_: normalized velocity, $\theta_{ij,k}\;=\;\underset{i,j,k}{L}(\frac{1}{v_{ij,k}})$, and function *L*(), which is used to normalize data, is defined as:

$$\underset{i}{L}\left(\psi_{ij}\right)=\frac{\psi_{ij}-\underset{i}{max}\left(\psi_{ij}\right)}{\underset{i}{max}\left(\psi_{ij}\right)-\underset{i}{min}\left(\psi_{ij}\right)}$$

τ_*ij*_: the “importance” of an interval;

δ_*ij*,*k*_: the discrepancy between the rank of link k given by interval j of day *i* and the overall ranks, i.e. *S* = {*s*_*k*_}; δ_*ij*,*k*_ corresponds to factor 2.

σ_*ij*,*k*_: the discrepancy between the rank of link k given by interval j at day *i* and those of other intervals; σ_*ij*,*k*_ corresponds to factor 3.

Thus the weight of a vote score is expressed as:

$$w_{ij,k}=f\left(\tau_{ij},\delta_{ij,k},\sigma_{ij,k}\right)$$

And *w*_*ij*,*k*_ should satisfy the relationship Σ_*i*,*j*_*w*_*ij*,*k*_ = 1.

For description purpose, we define:

$$\underset{i,j}{E}\left(\psi_{ij,k}\right)=\frac{\sum_{i,j}\psi_{ij,k}}{N\begin{array}{c} i,j\\ \psi_{ij,k} \end{array}}$$

The function is to calculate the mean of ψ_*ij*,*k*_ across all *i* and *j*. Ni,jψij,k denotes the total number of data.

#### Determination of τ_*ij*_

Note that during peak hours, more time is required to traverse unit distance, and thus we determine τ_*ij*_ as:

$$\tau_{ij}=\underset{i,j}{L}\left(\frac{1}{v_{ij}}\right)$$

Where *v*_*ij*_ is the mean velocity of all links during a specific interval *j* of day *i*:

$$v_{ij}=\underset{k}{E}\left(v_{ij,k}\right)=\frac{\sum_{k}v_{ij,k}}{N\begin{array}{c} k\\ v_{ij,k} \end{array}}$$

Nkvij,k is the number of velocity data during interval *j* of day *i*.

#### Determination of δ_*ij*,*k*_

It is assumed that the smaller the discrepancy δ_*ij*,*k*_ is, the greater the weight should be, hence, the we determine δ_*ij*,*k*_ as follows:

$$\delta_{ij,k}=\underset{k}{L}\left(\frac{1}{\left|\underset{k}{R}\left(\frac{1}{v_{ij,k}}\right)-s_{k}\right|}\right)$$

Where $\underset{k}{R}(\frac{1}{v_{ij,k}})$ is the rank of $\frac{1}{v_{ij,k}}$ for given *i* and *j*.

#### Determination of σ_*ij*,*k*_

For the score in interval *j* of day *i*, we assume it is better if its rank is closer to the average rank across, all time intervals. Hence the following function is utilized to determine σ_*ij*,*k*_:

$$\sigma_{ij,k}=\underset{i,j}{L}\left(\frac{1}{\left|\underset{k}{R}\left(\frac{1}{v_{ij,k}}\right)-\underset{i,j}{E}\left(\underset{k}{R}\left(\frac{1}{v_{ij,k}}\right)\right)\right|}\right)$$

Where $\underset{i,j}{E}(\underset{k}{R}(\frac{1}{v_{ij,k}}))$ is the mean of all $\underset{k}{R}(\frac{1}{v_{ij,k}})$ values across *i* and *j*.

Generally two forms of functions can be adopted for *w*_*ij*,*k*_ = *f*(τ_*ij*_, δ_*ij*,*k*_, σ_*ij*,*k*_).: additive and multiplicative. For the sake of simplicity, we use the following multiplicative form:

$$\begin{array}{c} w_{ij,k}=f\left(\tau_{ij},\delta_{ij,k},\sigma_{ij,k}\right)=\tau_{ij}\times \delta_{ij,k}\times \sigma_{ij,k}\\ =\underset{i,j}{L}\left(\frac{1}{v_{ij}}\right)\times \underset{k}{L}\left(\frac{1}{\left|\underset{k}{R}\left(\frac{1}{v_{ij,k}}\right)-s_{k}\right|}\right)\times \underset{i,j}{L}\left(\frac{1}{\left|\underset{k}{R}\left(\frac{1}{v_{ij,k}}\right)-\underset{i,j}{E}\left(\underset{k}{R}\left(\frac{1}{v_{ij,k}}\right)\right)\right|}\right) \end{array}$$

Once the weighting factors are derived, the score *s*_*k*_ can be obtained as:

$$s_{k}=\underset{k}{R}\left(\underset{i,j}{\sum }w_{ij,k}\times \frac{1}{v_{ij,k}}\right)$$

The steps for calculating link ranks can be summarized as follows:

1. Initialize the weights of all the votes, and making Σ_*i*,*j*_*w*_*ij*,*k*_ = 1, *∀i*, *j*, *k*
2. Compute the score of each link, $s_{k}\;=\;\underset{k}{R}(\sum_{i,j}w_{ij,k}\times \frac{1}{v_{ij,k}})$, *S* = {*s*_*k*_};
3. Normalize the score to the interval [0, 1];
4. Renew the weights, *w*_*ij*,*k*_ = *f*(τ_*ij*_, δ_*ij*,*k*_, σ_*ij*,*k*_);
5. If stop criteria is not satisfied, return to 2); end otherwise.

**Fig 8:**
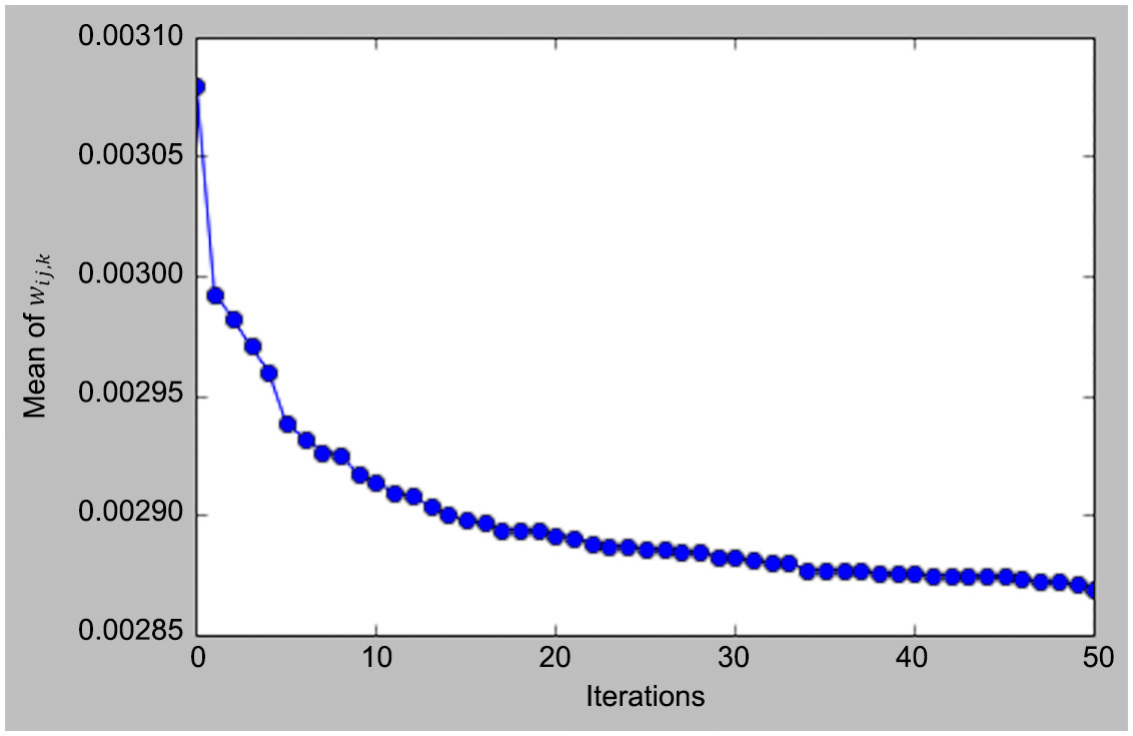
Convergence of mean of Σ_*i*,*j*,*k*_*w*_*ij*,*k*_.

Fig 8 shows the mean of *w*_*ij*,*k*_ during all the iterations at one voting. The data covers the entire 24 hours of day 2012-11-01. It can be seen that the algorithm gradually converges.

### Rolling Time Domain Method

**Fig 9:**
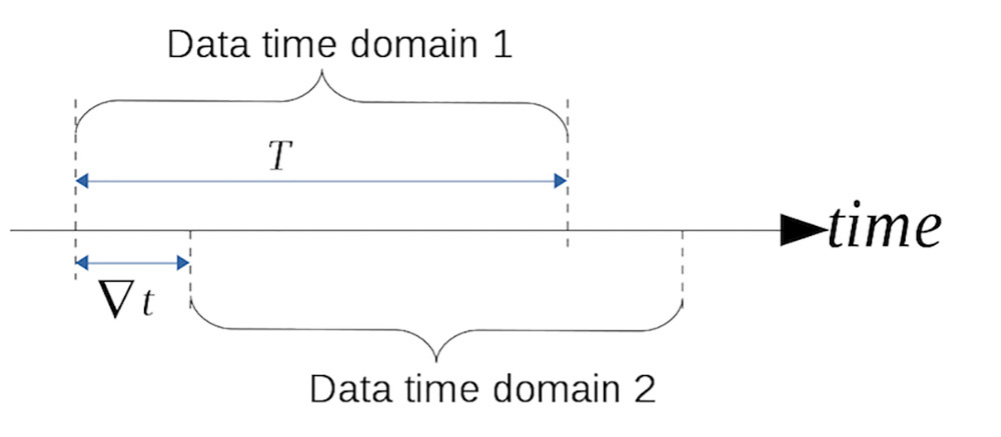
Rolling time domain method.

The lifecycle of a congestion area can be summarized as follows: at first, over-saturated queue forms at a single link. Then, this link will send as much flow as possible to its downstream links, which results in the over-saturation of downstream links. At the same time, the potential overflow queue will consume the capacity of the upstream intersection, which results in the over-saturation of upstream links. Thus a congestion area arises. The dispersion process is just the opposite. Thus a congestion area can be represented by a list of congestion link sets. Each set corresponds to a time interval and contains the congestion links during that time interval. Moreover, the links within the set form a connected sub-graph when we present the whole network as a graph. In order to obtain these sets, rolling time domain method is used. The idea is illustrated in Fig 9. There are two parameters, time domain length T and rolling step *∇t*. Each voting process will generate a ranking list of all the links. Then, we step forward to implement another voting, which will generate the voting result for the next time domain.

### Bottleneck Parameters

When the ranks are derived according to the velocities, some threshold can be set to select the top ranked links, which are more congested than others. These links comprise a sub-network. Within the sub-network, some links are connected while others are not, which means the sub-network can be composed of several components. We call these components the basic congestion unit. The congested components during adjacent time domains are relevant, since traffic states evolve from one time domain to the next. Various parameters can be defined based on the congested component generated, such as the size of the component, the center of the component, the average velocity of the component, etc. These parameters are used in the next section to facilitate the analysis.

**Fig 10:**
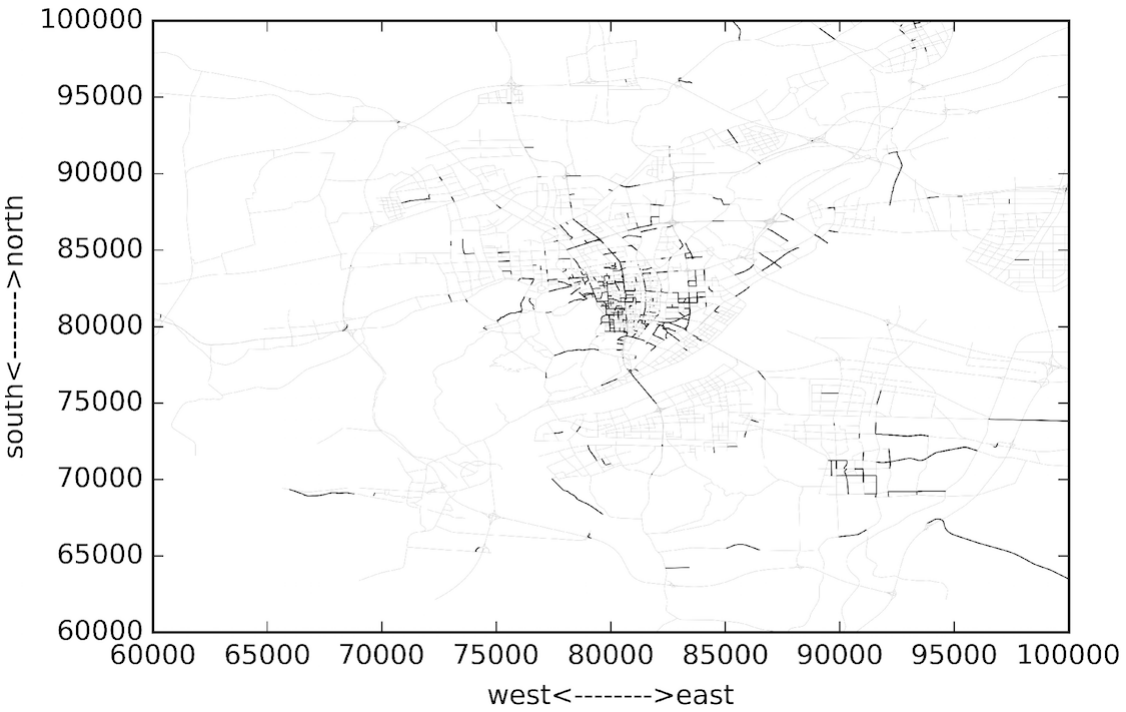
Top 1500 congested links for the whole day.

Fig 10 presents the top 1500 congested links of a whole day. The time domain length T is 24 hours. Gray lines denote uncongested links, while the thicker ones represent congested links. It can be observed that most congested links are located in or around the city center. Some other bottlenecks are scattered outside. While 24hours is too long to catch the variation of bottleneck areas, we set the time domain length T to one hour to get the bottleneck trend along time, since one hour is typical peak hour length thus allow us to analyze the dynamics of entering and exiting of congestion period. Longer time domain length leads to averaged result across time. Besides we set *∇t* to ten minutes to apply the time rolling voting, since 5 minutes will make the computation efficiency relative low and longer rolling step makes it hard to observe the evolution of bottleneck areas.

## Analysis Results

**Fig 11:**
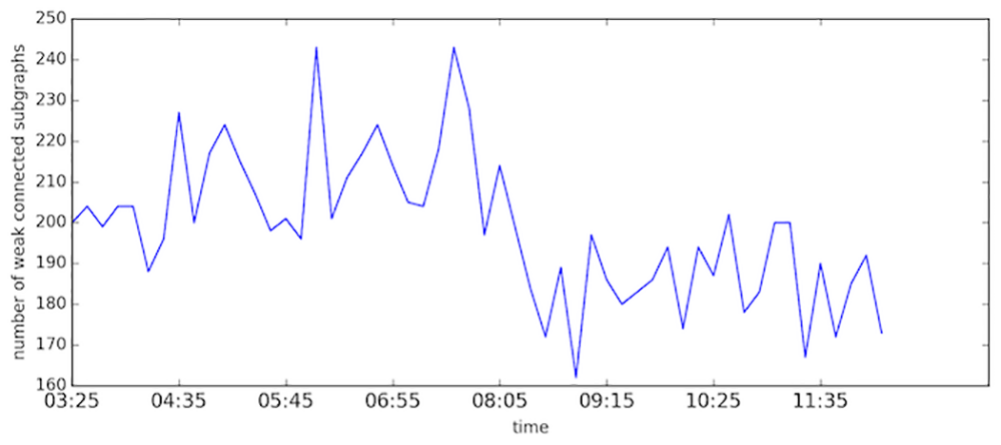
The number of components comprised by top 1500 links.

Here the rolling time domain length T and rolling step *∇t* are set to one hour and ten minutes, respectively. Fig 11 presents the numbers of congested components in different time domains. We examine the 1500 top ranked links for each time domain. These links comprise many isolated components. As congestion grows, some components expand and different components start to merge into larger components, which lead to a decrease in the number of components as shown in the figure. A sharp decrease is observed at about 08:00. Due to the stochastic nature of the transportation system, the number of components is not stable along the time horizon.

**Fig 12:**
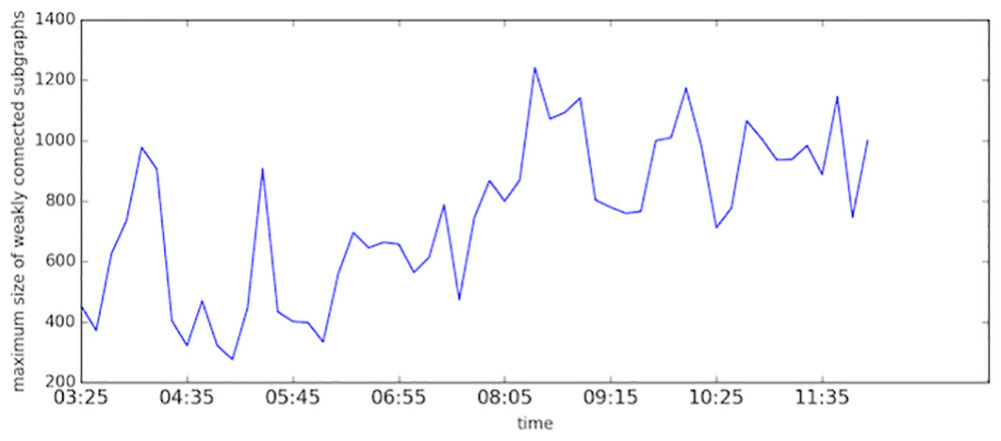
The size of the largest component in different time domains.

Fig 12 presents the size of the largest component in different time domains. The size means the number of links in the component. Generally, the size of the largest component increases when the morning peak approaches, which is reasonable since congestion area expands and different components start to merge. While even during peak hours, the size of the sub-network changes relatively.

**Fig 13:**
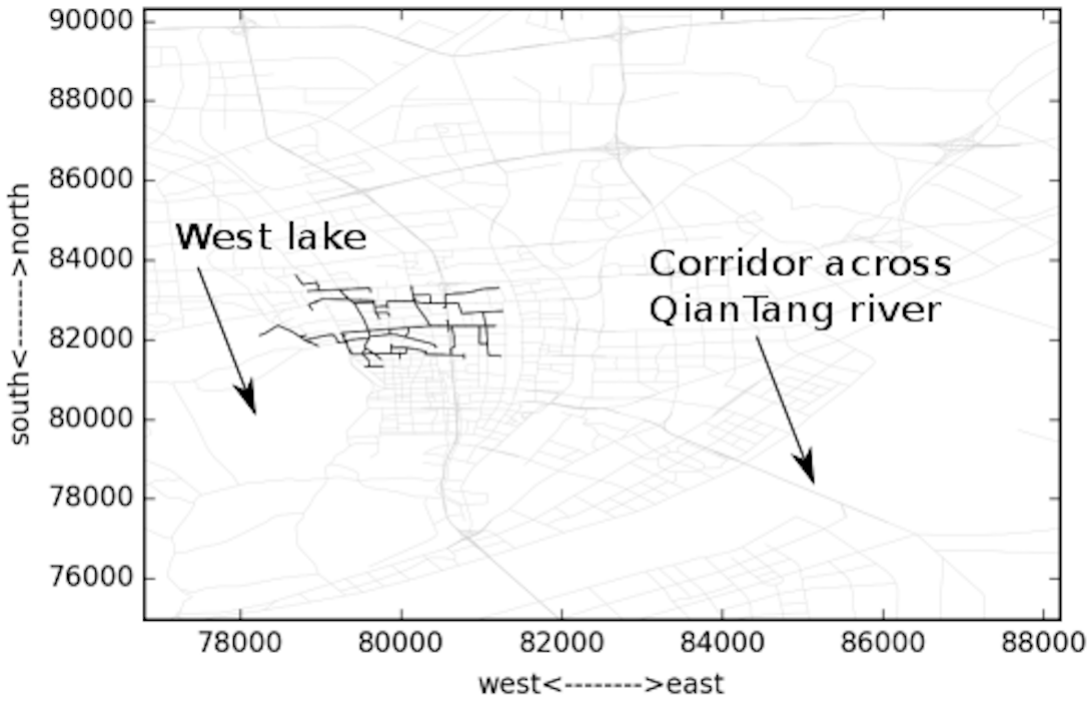
Largest congested component during 04:05~05:05.

**Fig 14:**
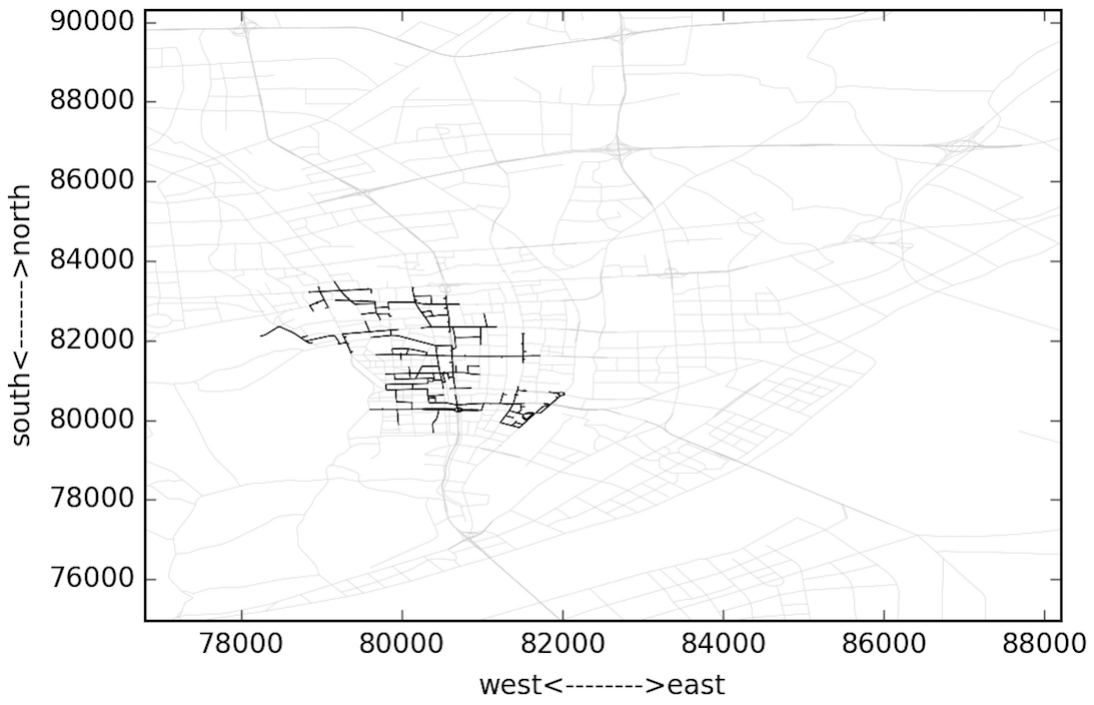
Largest congested component during 05:05~06:05.

**Fig 15:**
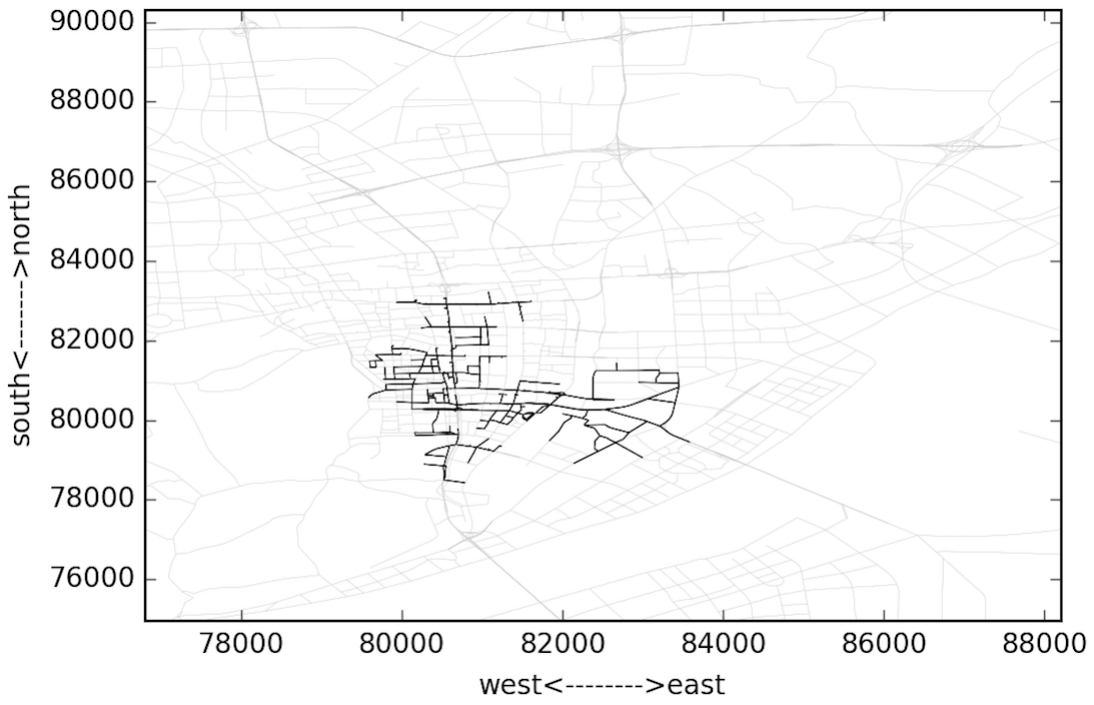
Largest congested component during 07:05~08:05.

**Fig 16:**
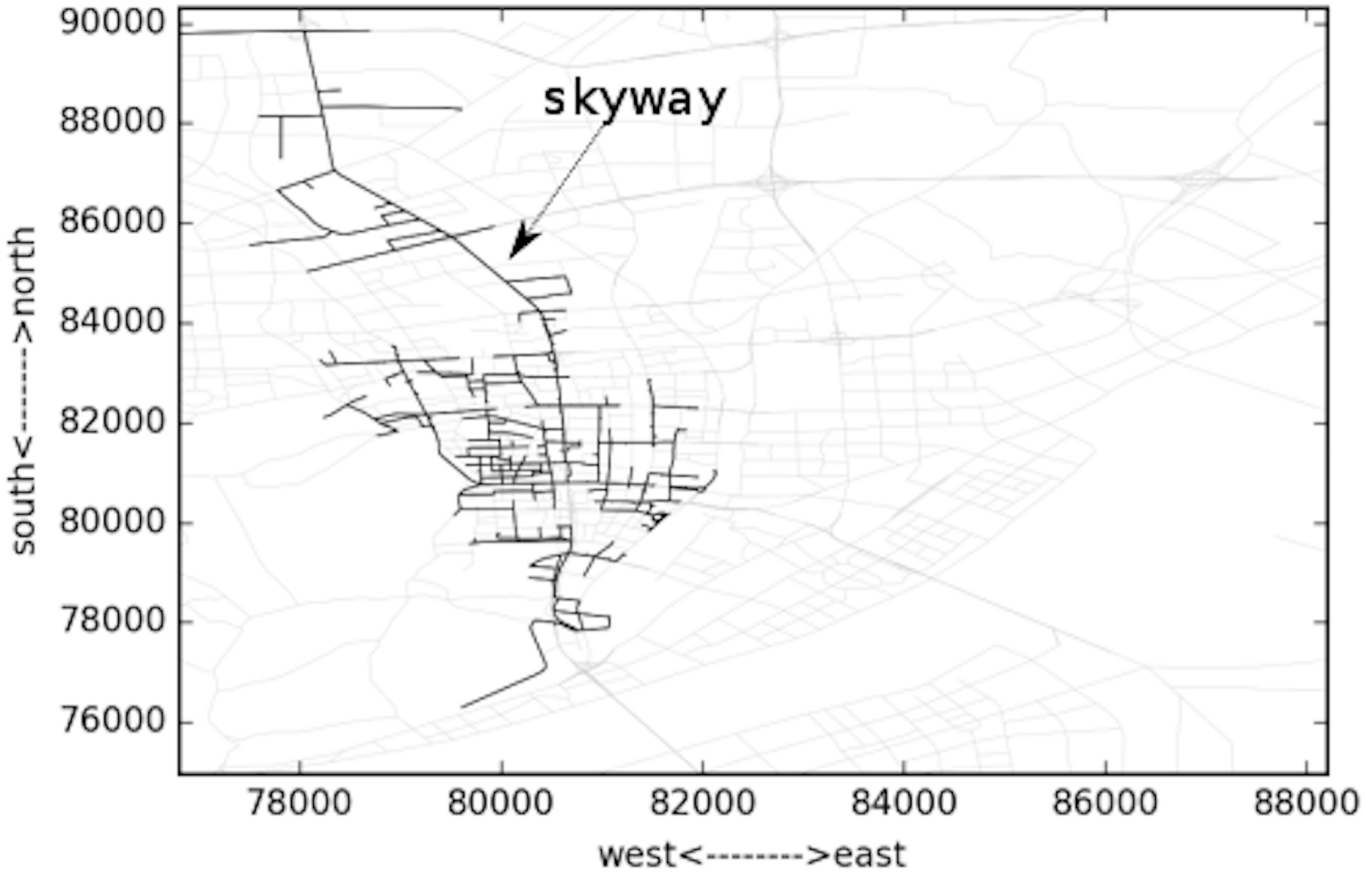
Largest congested component during 08:05~09:05.

Several typical time domains are selected to analyze the spatial changing of the congestion areas. These moments include 04:35, 05:53, 07:35 and 08:35. The largest congested components are shown in Figs 13–16. The links are all located to the east of “West Lake”, the most famous landscape in the city. This area is the traditional CBD area of the city, and the road density is very high. It can be observed in Fig 4 that the congestion peak in the morning is about 08:20. At first (04:35, Fig 13), the links in the component are mostly major roads, and only a few are branch roads. The density of the links in the component is lower compared to other three. This implies that most travelers select major roads during uncongested condition. As the rush time approaches, the congestion area changes spatially. At 07:35 as in Fig 15, the most congested area almost reaches the “XiXing bridge”, a major corridor that cross the QianTang river, which is indicated in Fig 13. In the morning, commuters enter the city through major roads, making these roads congested.

**Fig 17:**
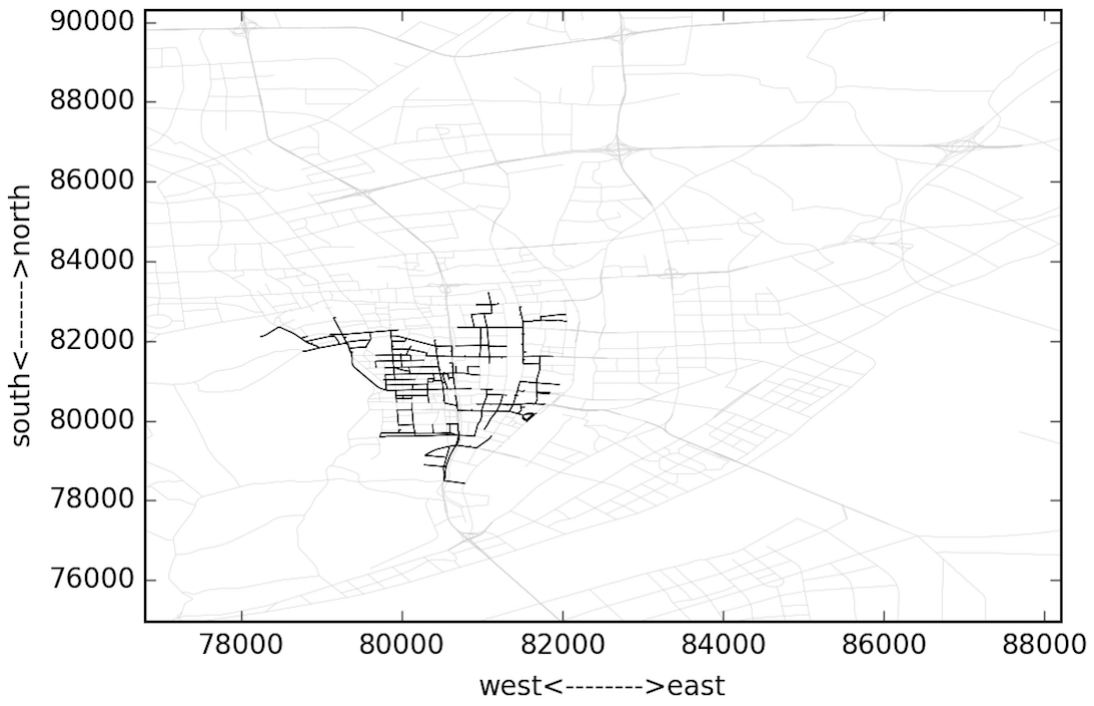
Largest congested component during 11:35~12:35.

From Fig 16, another important factor contributing to the spatial change of congestion area can be identified, which is the skyway almost across the entire city in the north-south direction. The skyway serves most of the relatively long trips. Because the capacity of the skyway is relatively high, and the interaction between this skyway and surface street network is controlled by ramp metering system, the velocity of the skyway is fast at the beginning, which makes it attractive to the long trips. Thus the flow on the skyway increases drastically. Once the congestion forms on the skyway, it will propagate very fast. We can see that the congestion almost covers the entire skyway. In the midday, at 12:05 in Fig 17, such congestion on the skyway does not exist.

## Conclusion

Urban traffic bottleneck has been the focus of many transportation studies, however, the methods and theories to analyze and eliminate bottlenecks are still lacking. Due to the development of modern data collection technologies, large scale traffic flow data becomes available and it provides great opportunities to construct urban bottleneck models. This paper takes advantage of new-technology based speed data and analyzed the dynamics of bottlenecks in the urban area of Hangzhou. The method of “voting” is introduced, where each velocity data is viewed as a score evaluated for the associated time interval, and the final score of a link is calculated based on the weighed sum of all the velocity data of this link. According to the scores of all the links, a link list can be created with more congested links ranked higher, thus the bottleneck links can be identified at the top of the list. The congestion areas, consists of connected bottleneck links, can be recognized based on the ranking result. Subsequently, by apply the rolling time horizon method, the evolution of the bottlenecks can be tracked explicitly. Numerical examples have been provided to demonstrate the proposed data analysis approach.

However, identification of bottleneck is the first step towards operation improvements. In order to make decision on improvement plans, deep understanding of sensitivity of road capacity and network demand to bottleneck dynamics is required. Furthermore, the overall evaluation of the congestion, namely the supply-demand structure should be carried out before the decision, which can be realized based on the results of this research.

## Supporting Information

S1 File Original tabular data of velocity in the paper. (ZIP)
